# Comparison of two different frailty scales in the longitudinal Swedish Adoption/Twin Study of Aging (SATSA)

**DOI:** 10.1177/14034948211059958

**Published:** 2021-12-14

**Authors:** Alexandra M. Wennberg, Weiyao Yin, Fang Fang, Nancy L. Pedersen, Sara Hägg, Juulia Jylhävä, Karin Modig

**Affiliations:** 1 Unit of Epidemiology, Institute of Environmental Medicine, Karolinska Institutet, Sweden; 2 Unit of Integrative Epidemiology, Institute of Environmental Medicine, Karolinska Institutet, Sweden; 3 The Department of Medical Epidemiology and Biostatistics, Karolinska Institutet, Sweden

**Keywords:** Frailty, registries, Sweden, mortality

## Abstract

**Aims:**

Although up to 25% of older adults are frail, assessing frailty can be difficult, especially in registry data. This study evaluated the utility of a code-based frailty score in registry data by comparing it to a gold-standard frailty score to understand how frailty can be quantified in population data and perhaps better addressed in healthcare.

**Methods:**

We compared the Hospital Frailty Risk Score (HFRS), a frailty measure based on 109 ICD codes, to a modified version of the Frailty Index (FI) Frailty Index (FI), a self-report frailty measure, and their associations with all-cause mortality both cross-sectionally and longitudinally (follow-up = 36 years) in a Swedish cohort study (*n* = 1368).

**Results:**

The FI and HFRS were weakly correlated (rho = 0.11, *p* < 0.001). Twenty-two percent (*n* = 297) of participants were considered frail based on published cut-offs of either measure. Only 3% (*n* = 35) of participants were classified as frail by both measures; 4% (*n* = 60) of participants were considered frail by only the HFRS; and 15% (*n* = 202) of participants were considered frail based only on the FI. Frailty as measured by the HFRS showed greater variance and no clear increase or decrease with age, while frailty as measured by the FI increased steadily with age. In adjusted Cox proportional hazard models, baseline HFRS frailty (HR = 1.17, 95% CI 0.92, 1.49) was not statistically significantly associated with mortality, while FI frailty was (HR = 2.89, 95% CI 1.61, 2.23). These associations were modified by age and sex.

**Conclusions:**

**The HFRS may not capture the full spectrum of frailty among community-dwelling individuals, particularly at younger ages, in Swedish registry data.**

## Background

Frailty is a chronic condition that impacts functioning and quality of life and affects 12 to 24% of older adults [1]. It is characterized by the inability to respond to chronic or acute stressors, resulting from a decrease in physical or biological reserve and the subsequent inability to maintain homeostasis [2]. In the last year of life, frailty is the most common condition leading to death, more common than organ failure, cancer, or dementia [3]. However, frailty is difficult to reliably recognize because of the myriad of ways it presents. Aging and the health of older adults are heterogeneous, often defined by multimorbidity and polypharmacy [4]. Although multimorbidity and frailty are frequently thought of in parallel, the modern healthcare system is designed around a single-disease approach, so this model will not serve the growing aging population with its varied, complex healthcare needs. Understanding frailty patterns in populations is necessary to shift healthcare systems to better serve older adults.

Different frailty indices have been developed based on different concepts of frailty and evaluated in diverse samples, varying by age, sex, or location (e.g., hospital vs. research sample) [5, 6]. Frailty is measured by assessing physical phenotype (i.e., weakness, slowness, exhaustion, low physical activity, and unintentional weight loss) or cumulative deficits. The Rockwood Frailty Index (FI) is based on a deficit accumulation framework and uses a combination of symptoms, diseases, conditions, and disability to predict frailty [7]. It is particularly good at predicting outcomes like all-cause and cardiovascular disease mortality [8], as well as stratifying risk in populations [9]. It is better than other frailty metrics at identifying frail individuals and risk of associated outcomes at the healthier (less frail) end of the spectrum [10, 11], making it more suitable for younger adults. Illustrating this, we have previously shown that there is a steady increase in FI score up until age 65, after which there is a dramatic increase [12]. Moreover, in this work, it was shown that FI score is associated with mortality, especially among younger (<65) adults, and that this association was more robust among women as compared to men [8]. The Hospital Frailty Risk Score (HFRS) is also based on the cumulative deficit framework and has been validated previously to predict adverse outcomes in various settings [13, 14]. More recently, it was found that, although HFRS was developed for older individuals, it was more strongly associated with COVID-19-related mortality in individuals <75 [15].

Although past studies have compared frailty measures and examined their ability to predict adverse outcomes, many of these studies have been limited by relatively small sample sizes and cross-sectional designs. To expand upon this earlier research, we compared the overlap between the FI and the HFRS, as well as their respective association with all-cause mortality, in the Swedish Adoption/Twin Study of Aging (SATSA). The FI is an established measure of frailty based on multiple aspects of health assessed in interview or through self-report, whereas the HFRS is based on International Classification of Diseases (ICD) codes and can be derived from registry data. Population registers used for research in many countries, especially the Nordic countries, make it possible to link data on an individual level and present a unique opportunity to examine geriatric conditions, including frailty, at a population level with essentially zero attrition. However, registry data rely on hospital codes and clinical data. Therefore, understanding how best to assess frailty in these data is a necessary first step to consider how we can best leverage it to understand patterns of, risk factors for, and healthcare services directed at frailty on the population level.

## Methods

### Participants

SATSA is a longitudinal cohort study of community-dwelling same-sex twins, some of whom were raised together whereas others were separated before age 11 [16]. SATSA data were collected from 1984 to 2014 in nine questionnaires (Q) and 10 in-person testing (IPT) waves (Supplemental Figure 1). It has rich data on medical, lifestyle, psychosocial, and socioeconomic variables. Only individuals ⩾50 were invited to participate in IPTs, and new participants were enrolled only until the fifth IPT. The present study includes data from 1430 participants who participated in both Qs and IPTs, if eligible. The current study is part of a research program that is performed in accordance with the Declaration of Helsinki and was approved by the ethics board of Karolinska Institutet. All participants provided informed consent.

### Frailty measures: SATSA Frailty Index

At SATSA waves, participants answered questions related to their health status, and these answers were used to create the SATSA FI score for each individual. Participants answered these questions at all of the SATSA waves except Q1, IPT1, IPT4, and Q6 (Supplemental Figure 1). The FI score in SATSA is comprised of 42 self-reported health deficits (Supplemental Table 1), including diseases, symptoms, mood, and activities of daily living. A detailed description is provided by Raymond and colleagues [12], although, briefly, deficits are summed and divided by 42 (e.g., a person who has 10 deficits has a score of 0.24, 10/42 = 0.24) at each wave [17]. A person with a score of 0.21 or higher is considered frail.

### Frailty measures: Hospital Frailty Risk Score

A version of the HFRS was additionally used to assess frailty [14]. This scale calculates the sum of 109 hospital-based ICD-10 codes weighted from 0.1 to 7.1, which we translated from the international version of the ICD-10 to the Swedish version of the ICD-10, -9, and -8 (Supplemental Table 2). Hospitalization and outpatient care (from 2001 onwards) were ascertained using the National Patient Register for the years of the SATSA waves and summed over a period of 3 years – the year of the SATSA wave and 1 year before and after. The HFRS was scored at each year; scores 0–5 were considered low risk of frailty, 5–15 moderate risk, and >15 high risk [14]. Individuals with missing hospital data (i.e., who had not been hospitalized that year) were coded as having a 0 for the HFRS.

### Sum of illnesses and number of medications

At the IPTs, participants also self-reported the medications they were taking and completed a questionnaire about their known illnesses. The medications were summed to create a polypharmacy score. Drugs with the same Anatomical Therapeutic Chemical (ATC) classification at ATC4 level were coded as the same type. The sum of illnesses measure includes 13 conditions (i.e., cardiovascular, respiratory, musculoskeletal, allergy, skin problems, central nervous system-related disorder, eye problems, metabolic type disorder, gastrointestinal tract disorder, urological disorder, cancer/leukemia, ear problems, and disorders of female reproductive organs) [16].

### All-cause mortality

We additionally examined all-cause death as an outcome. Death and date of death were determined by record linkage to the National Population Register through June 24, 2020. This resulted in a 36-year follow-up period in this analysis.

### Statistical analysis

Participant baseline characteristics were summarized as mean and standard deviation (SD) or number and percentage. We stratified participants using the frailty cut-points for the HFRS (>5) and FI (>0.21), and summarized characteristics based on the following four groups: (1) frail by both metrics, (2) frail by HFRS but not FI, (3) frail by FI but not HFRS, and (4) not frail. The overlap between frailty as defined by these scores and the median of the sum of illnesses (>6) was plotted in Venn diagrams. We further used Spearman’s rank correlation analysis to examine the correlation between continuous HFRS and FI scores. To visualize patterns of the HFRS and FI both cross-sectionally and longitudinally, we plotted the respective median (interquartile range) scores as panel data using age as the timescale. Finally, we used Cox proportional hazard models to examine the association between frailty (defined using cut points) and mortality, adjusting for age and sex in Model 1. We specified frailty (0, 1), defined by a score of greater than 5 on the HFRS and greater than 0.21 on the FI, as the independent variables in the models and all-cause mortality as the dependent variable. We additionally investigated continuous scores for sum of illnesses and number of medications as predictors of all-cause mortality. Proportional hazards assumptions, assessed with the proportional hazards plot function in Stata, were met in all models. In sensitivity analyses, we examined sex and age (<75 vs. >75 years, often used in the literature) as effect modifiers.

## Results

**Figure 1.**
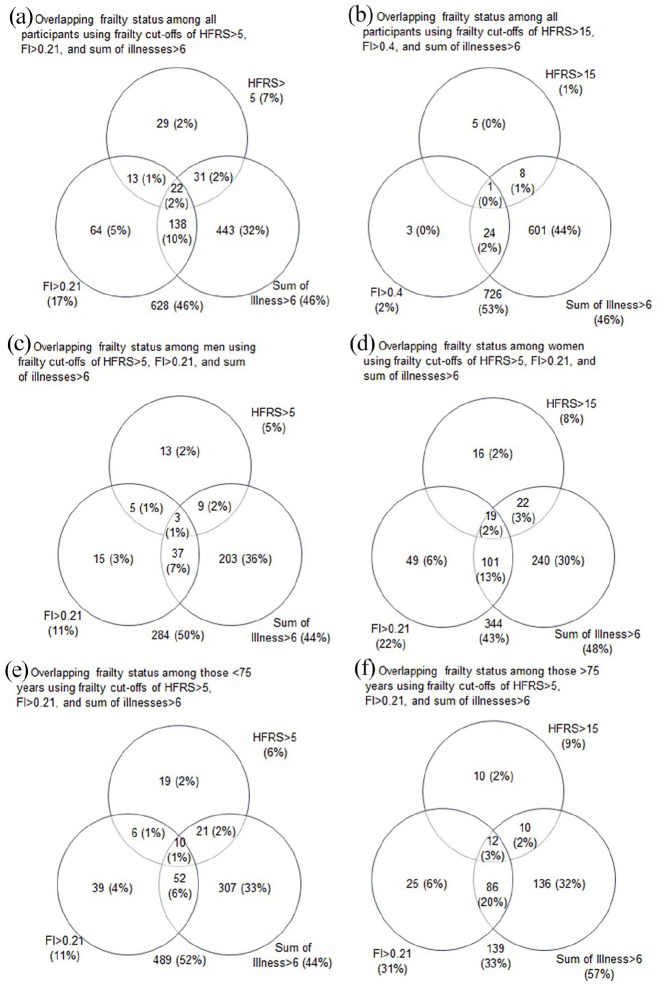
Venn diagrams of overlap of frail individual based on the respective cut-points. Diagrams represent all participants (a, b) and participants stratified by sex (c, d) and age (e, f).

**Figure 2.**
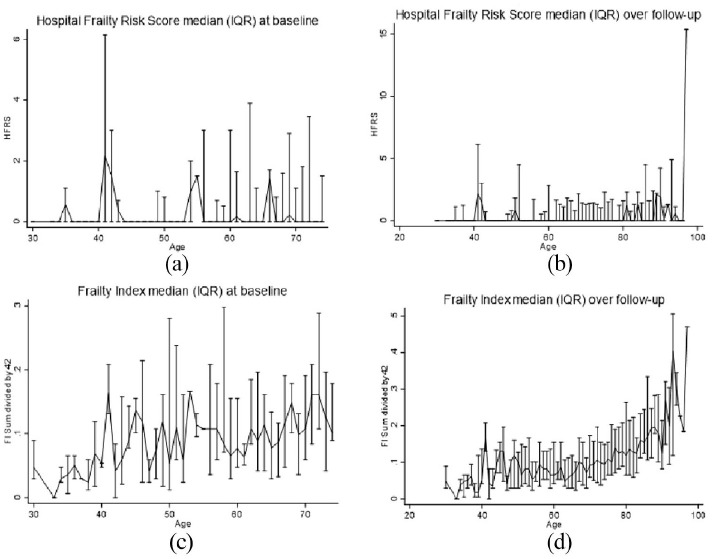
Distributions of continuous HFRS and FI scores (median (IQR)) at baseline (a, c) and longitudinally (b, d) using repeated measures, using age as a timescale.

This study included 1368 SATSA participants followed for an average (SD) of 6.1 (4.2) waves (Table I). At baseline, slightly less than half (42%) were men, and the average age was 68.0 (12.3, range = 29–96) years. The mean FI score was 0.12 (0.10), the mean HFRS score was 0.59 (3.0), and these continuous measures were weakly but statistically significantly correlated with each other (rho = 0.11, *p* < 0.001). In participants >75, the correlation was less robust (rho = 0.07, *p* = 0.061) compared with those <75 (rho = 0.20, *p* < 0.001). Using the cut points for the HFRS (>5) and FI (>0.21), a total of 22% (*n* = 297) of participants were considered frail at baseline. Only 3% (*n* = 35) of participants were classified as frail by both measures. Similarly, 4% (*n* = 60) of participants were considered frail by the HFRS but not by the FI. By comparison, 15% (*n* = 202) of participants were considered frail based on the FI but not the HFRS. This lack of overlap (Figure 1) was driven mainly by the HFRS and its low sensitivity and did not change when stratified by age or sex. Across age, the HFRS showed greater variance but no clear increase or decrease, except at the oldest ages (Figure 2). By comparison, FI showed an increase with age, with a steadier increase after age 50.

**Table I.** Participant characteristics at first SATSA wave, *N* (%) or mean (SD).

	All	FI-frail/HFRS-frail	FI-nonfrail/HFRS-nonfrail	HFRS-frail/FI-nonfrail	FI-frail/HFRS-nonfrail
*N*	1368	35	1071	60	202
Male, *N* (%)	569 (42)	8 (23)	487 (46)	22 (37)	150 (74)
Age, year	68.0 (12.3)	74.7 (9.4)	66.5 (12.1)	69.3 (10.7)	74.2 (11.5)
SATSA waves	6.1 (4.2)	2.9 (2.4)	6.7 (4.2)	5.9 (3.9)	3.7 (2.8)
Sum of illnesses	3.4 (2.0)	5.1 (1.8)	2.9 (1.6)	2.9 (1.8)	6.0 (2.0)
Number medications	2.5 (1.8)	3.2 (1.8)	2.3 (1.6)	2.1 (1.5)	3.9 (2.4)
Sum FI (total deficits)	5.0 (4.1)	12.9 (3.5)	3.5 (2.2)	4.0 (2.3)	12.2 (3.4)
FI (sum divided by 42)	0.12 (0.10)	0.30 (0.08)	0.08 (0.05)	0.10 (0.05)	0.29 (0.08)
FI (median (IQR))	0.10 (0.05, 0.16)	0.30 (0.23, 0.36)	0.08 (0.04, 0.12)	0.09 (0.05, 0.15)	0.27 (0.23, 0.33)
HFRS	0.59 (3.0)	6.5 (11.9)	0.25 (0.70)	3.8 (8.9)	0.41 (0.96)
HFRS categories					
0–5	1273 (93)	–	1071 (100)	–	202 (100)
5–15	81 (6)	27 (77)	–	54 (90)	–
>15	14 (1)	8 (23)	–	6 (10)	–
Number of deaths, *N* (%)	924 (68)	33 (94)	670 (63)	41 (68)	180 (89)

* Mean and median of FI presented because it is a skewed measure.

Longitudinally, we observed much the same pattern. The HFRS did not show a substantial increase with age (Figure 2), while FI showed a steady increase with more variability at younger ages and a sharper increase starting at age 60. In Cox proportional hazard models adjusted for age and sex, baseline HFRS (HR = 1.17, 95% CI 0.92, 1.49) was not statistically significantly associated with mortality, while the FI was statistically significantly associated with mortality (HR = 1.89, 95% CI 1.61, 2.23) (Table II). As surrogates of frailty and poorer health, we examined whether sum of illnesses score and number of medications were associated with mortality in the subset of participants for whom these data were available. Both sum of illnesses (HR = 1.05, 95% CI 1.01, 1.09) and number of medications (HR = 1.10, 95% CI 1.05, 1.15) were statistically significantly associated with mortality. In sensitivity analyses stratified by sex, FI was more strongly associated with mortality in women (HR = 2.03, 95% CI 1.66, 2.48) than in men (HR = 1.69, 95% CI 1.26, 2.25) (Table III). By comparison, the HFRS was not associated with mortality in either men (HR = 1.08, 95% CI 0.71, 1.63) or women (HR = 1.23, 95% CI 0.92, 1.65). In sensitivity analyses stratified by age, the FI was more strongly associated with mortality in those younger than 75 (HR = 2.16 95% CI 1.71, 2.73) than those older than 75 (HR = 1.83, 95% CI 1.46, 2.29). The HFRS was not statistically significantly associated with mortality in either the younger (HR = 1.18, 95% CI 0.85, 1.64) or older (HR = 1.17, 95% CI 0.83, 1.65) group (Table III).

**Table II.** Cox proportional hazard estimates for the association between HFRS/FI frailty and mortality among all participants.

All participants	HFRS	FI	Sum of Illnesses (*n* = 888)	Number of medications (*n* = 648)
	HR (95% CI)	HR (95% CI)	HR (95% CI)	HR (95% CI)
Frailty measure	1.17 (0.92, 1.49)	1.89 (1.61, 2.23)	1.05 (1.01, 1.09)	1.10 (1.05, 1.15)
Sex	0.61 (0.53, 0.70)	0.57 (0.50, 0.65)	0.61 (0.52, 0.71)	0.56 (1.05, 1.15)
Age	1.11 (1.10, 1.12)	1.11 (1.10, 1.12)	1.11 (1.10, 1.12)	1.12 (1.11, 1.14)

**Table III:** Cox proportional hazard estimates for the association between HFRS/FI frailty and mortality stratified by sex and age group.

Sex stratified	Men (*n* = 569)		Women (*n* = 799)	
	HFRS	FI	HFRS	FI
Frailty measure	1.08 (0.71, 1.63)	1.69 (1.26, 2.25)	1.23 (0.92, 1.65)	2.03 (1.66, 2.48)
Age	1.10 (1.09, 1.12)	1.10 (1.09, 1.11)	1.12 (1.11, 1.13)	1.12 (1.10, 1.13)
Age stratified	<75 years old (*n* = 943)		>75 years old (*n* = 425)	
	HFRS	FI	HFRS	FI
Frailty measure	1.18 (0.85, 1.64)	2.16 (1.71, 2.73)	1.17 (0.83, 1.65)	1.83 (1.46, 2.29)
Sex	0.62 (0.53, 0.74)	0.57 (0.48, 0.68)	0.58 (0.47, 0.72)	0.55 (0.44, 0.69)
Age	1.12 (1.11, 1.13)	1.12 (1.11, 1.13)	1.11 (1.08, 1.14)	1.10 (1.07, 1.12)

## Discussion

Our findings suggest that although both the FI and HFRS are associated with risk of mortality, the FI is more sensitive to capturing frailty and associated risk of mortality among younger individuals, who are likely to be less frail. The steady increase in FI score up until age 60, with a sharper rise thereafter, echoes a previous finding in this cohort [12]. By contrast, the more variable pattern of the HFRS across age likely impacted the association with mortality. Our findings suggest that the HFRS is not as sensitive and is more variable than the FI; therefore, it may not detect risk of frailty in a sample of relatively healthy community-dwelling adults. We found very little overlap of frail and non-frail individuals at any given age between the FI and HFRS. Additionally, although the FI increased across age in the cohort, the HFRS did not. These differences suggest that the two measures capture different aspects of health. Despite the variability in the HFRS, effect sizes suggest it is associated with mortality and may be relevant for both clinical and research applications when using population registry data.

Previous longitudinal studies using smaller samples and shorter follow-up to compare frailty indices have shown similar results. A year-long study that compared six frailty indices, including the FI, found that higher frailty scores were associated with mortality independent of age and sex, among adults aged 65 to 82 at baseline [18]. This association has also been shown in studies of hospitalized patients [19, 20] and those on geriatric wards [21-23]. The long (36 year) follow-up period in a sample with a large age range allowed us to broadly consider the potential predictive abilities of the FI and HFRS in this study. Our study and others [24] found that the FI was most strongly associated with mortality in participants under 75 years, as compared to older individuals. This finding likely reflects that having frailty at a younger age is representative of more severe health issues and linked to accelerated trajectories to death [3]. These factors may help to explain why the HFRS was not as robust as the FI at assessing frailty and associated mortality. However, notably, there were few participants in this study with an HFRS of ⩾15 (the highest category), perhaps because primary care data were not available. This likely limited the sensitivity and power, as exemplified by the wide confidence intervals. Inclusion of this data would provide information about less acute or serious health events and conditions, likely improving the ability of the HFRS to detect frailty and associated mortality. However, the hazard ratios associated with the HFRS were consistently greater than 1, suggesting that the association between HFRS and all-cause mortality might indeed exist, even if it did not reach statistical significance.

Our findings support the hypothesis that the HFRS is more of a snapshot and event-determined measure, while the FI better depicts the more subtle or subjective aspects of frailty that build over time. Although the HFRS may provide a snapshot of significant health events, it may not capture the full health picture, particularly in community-dwelling adults. This may be because the HFRS was developed in persons aged ⩾75 with high resource use in secondary care. Therefore, it may be necessary to perform subgroup analyses with different cut-offs to more accurately apply the score in different settings [25]. By comparison, the FI may provide a more encompassing view of health, and – even at lower scores (representing lower levels of frailty) – may better predict longer term risk of mortality. This further supports the idea that an integrated conceptualization of frailty that takes physical conditions, as well as psychological, cognitive, social, and functional components of health into consideration, is most useful in clinical and research settings [22].

This study has multiple strengths, including the longitudinal design with a long follow-up period and low attrition. However, the sample size, while larger than other similar studies, may still be too limited, particularly in sensitivity analyses, stratifying by multiple subgroups. Second, the SATSA study design meant that only participants aged 50 and older participated in IPTs, thus limiting the number of times frailty was assessed in younger participants. Past studies have shown that frailty at younger ages is more strongly associated with mortality [8], so it is important to consider and monitor even younger individuals exhibiting risk factors or signs of frailty. Additionally, the FI is a measure of frailty based on self-report, so there may be reporting bias. However, the FI is a gold-standard measure of frailty and has shown excellent validity and reliability with non-self-report frailty measures [26]. Further, the HFRS does not include external ICD codes (i.e., secondary codes that capture specific details about an injury or health event), so the results may be attenuated toward the null, but our other work has shown this does not impact frailty-related associations [27]. Finally, this study was able to assess only two measures of frailty, although other studies have compared multiple measures [22], which may allow for a more expansive comparison.

Together the findings that frailty is most strongly associated with mortality at younger ages and the evidence showing that it increases across decades indicate that frailty interventions might be most effective if implemented before the sixth decade [12]. Indeed, because frailty is both a risk factor (e.g., for mortality) [3] and an outcome, interventions to prevent and address it are critical. However, the first step is to understand it, and registry data are important resources for mapping population trends. Although scales specifically designed to quickly assess frailty at primary care appointments have been created [18, 28], these do not translate to codes, limiting what may be ascertained from national registry databases that compile ICD diagnoses. Considering whether the HFRS can be used to accurately assess frailty in Nordic registries is of great importance for geriatric epidemiological studies in this context. Fine-tuning tools for assessing the spectrum of frailty in these datasets would be helpful for measuring the impact of frailty, both as a predictor and an outcome. This study represents an initial step in assessing frailty at the population level, with the eventual goal of understanding patterns of, risk factors for, and healthcare directed at frailty.

## Supplemental Material

sj-docx-1-sjp-10.1177_14034948211059958 – Supplemental material for Comparison of two different frailty scales in the longitudinal Swedish Adoption/Twin Study of Aging (SATSA) Supplemental material, sj-docx-1-sjp-10.1177_14034948211059958 for Comparison of two different frailty scales in the longitudinal Swedish Adoption/Twin Study of Aging (SATSA) by Alexandra M. Wennberg, Weiyao Yin, Fang Fang, Nancy L. Pedersen, Sara Hägg, Juulia Jylhävä and Karin Modig in Scandinavian Journal of Public Health

sj-png-2-sjp-10.1177_14034948211059958 – Supplemental material for Comparison of two different frailty scales in the longitudinal Swedish Adoption/Twin Study of Aging (SATSA) Supplemental material, sj-png-2-sjp-10.1177_14034948211059958 for Comparison of two different frailty scales in the longitudinal Swedish Adoption/Twin Study of Aging (SATSA) by Alexandra M. Wennberg, Weiyao Yin, Fang Fang, Nancy L. Pedersen, Sara Hägg, Jylhävä and Karin Modig in Scandinavian Journal of Public Health
