# Supplementary material for: Comparison of two different frailty scales in the longitudinal Swedish Adoption/Twin Study of Aging (SATSA)
Source: Scand J Public Health. 2021 Dec 14;51(4):587–94. doi: 10.1177/14034948211059958 (PMC10265292; doi:10.1177/14034948211059958)
Supplement: sj-docx-1-sjp-10.1177_14034948211059958 – Supplemental material for Comparison of two different frailty scales in the longitudinal Swedish Adoption/Twin Study of Aging (SATSA) [file sj-docx-1-sjp-10.1177_14034948211059958.docx]

| Supplementary Table 1. SATSA frailty index variables and scoring | |
| --- | --- |
| Variable/condition | Scoring |
| Allergies/allergic manifestations | No=0, Yes=1 |
| Anemia | No=0, Yes=1 |
| Arthritis | No=0, Yes=1 |
| Asthma | No=0, Yes=1 |
| Cancer or leukemia | No=0, Yes=1 |
| Cataracts | No=0, Yes=1 |
| Cerebral haemorrhage or blood clot in brain | No=0, Yes=1 |
| Chest pain | No=0, Yes=1 |
| Chronic bronchitis or emphysema | No=0, Yes=1 |
| Circulation problems in arms or legs | No=0, Yes=1 |
| Diabetes | No=0, Yes=1 |
| Dizziness | No=0, Yes=1 |
| Gastric ulcer | No=0, Yes=1 |
| Goiter or other gland problems | No=0, Yes=1 |
| Heart failure | No=0, Yes=1 |
| Hypertension | No=0, Yes=1 |
| Kidney disease | No=0, Yes=1 |
| Osteoporosis | No=0, Yes=1 |
| Persistent cough | No=0, Yes=1 |
| Rheumatoid arthritis | No=0, Yes=1 |
| Sciatica | No=0, Yes=1 |
| Usually feels tired | No=0, Yes=1 |
| Consider oneself happy and carefree | No=0, Yes=1 |
| Hearing status | Perfect = 0, Good = 0.25, Pretty good = 0.5, Bad = 0.75, Deaf or almost deaf = 1 |
| Vision status | Perfect = 0, Good = 0.25, Pretty good = 0.5, Bad = 0.75, Deaf or almost deaf = 2 |
| Feeling depressed^1^ | Never, almost never, rather seldom = 0; Quite often, almost always, always = 1 |
| Feeling lonely^1^ | Never, almost never, rather seldom = 0; Quite often, almost always, always = 2 |
| Housework^2^ | No problem = 0, Needs help = 0.5, Cannot = 1 |
| Grocery shopping^2^ | No problem = 0, Needs help = 0.5, Cannot = 1 |
| Manage medications^2^ | No problem = 0, Needs help = 0.5, Cannot = 1 |
| Manage money^2^ | No problem = 0, Needs help = 0.5, Cannot = 1 |
| Prepare meals^2^ | No problem = 0, Needs help = 0.5, Cannot = 1 |
| Travel further distances^2^ | No problem = 0, Needs help = 0.5, Cannot = 1 |
| Use telephone^2^ | No problem = 0, Needs help = 0.5, Cannot = 1 |
| Dress and undress^3^ | No problem = 0, Needs help = 0.5, Cannot = 1 |
| Get in and out of bed^3^ | No problem = 0, Needs help = 0.5, Cannot = 1 |
| Self-grooming^3^ | No problem = 0, Needs help = 0.5, Cannot = 1 |
| Shower and bathe^3^ | No problem = 0, Needs help = 0.5, Cannot = 1 |
| Walking^3^ | No problem = 0, Needs help = 0.5, Cannot = 1 |
| Trouble getting to toilet in time^3^ | No = 0, Yes / I have a catheter or colostomy = 1 |
| Self-reported general health | Good=0, Mediocre=0.5, Bad=1 |
| Health prevents from doing things normally would like to do | No=0, Somewhat=0.5, Yes=1 |
| Note: ^1^from the Center for Epidemiological Studies - Depression Scale (CES-D) (29), ^2^from the instrument of Instrumental Activities of Daily Living (30) and ^3^ from the instrument of Basic Activities of Daily Living (30). | |

[29] Chamberlain AM, St Sauver JL, Jacobson DJ, et al. Social and behavioural factors associated with frailty trajectories in a population-based cohort of older adults. *BMJ Open* 2016;6:e011410.

[30] Chamberlain AM, Finney Rutten LJ, Manemann SM, et al. Frailty trajectories in an elderly population-based cohort. *J Am Geriatr Soc* 2016;64:285–92.

| Supplementary Table 2. Swedish ICD 9 and 10 codes used to create the HFRS | | | |
| --- | --- | --- | --- |
| ICD-8-9 | ICD-10 | ICD DESCRIPTION | WEIGHT |
| 331A | F00 | Dementia in Alzheimer's disease | 7.1 |
| 342X | G81 | Hemiplegia | 4.4 |
| 331A | G30 | Alzheimer's disease | 4.0 |
| 438 | I69 | Sequelae of cerebrovascular disease | 3.7 |
| 781X | R29 | Other symptoms and signs involving the nervous and musculoskeletal systems | 3.6 |
| 596X | N39 | Other disorders of urinary system (includes urinary tract infection and urinary incontinence) | 3.2 |
| 293 | F05 | Delirium, not induced by alcohol and other psychoactive substances | 3.2 |
| 910W | S00 | Superficial injury of head | 3.2 |
| 599H | R31 | Unspecified haematuria | 3.0 |
| 041 | B96 | Other bacterial agents as the cause of diseases classified to other chapters | 2.9 |
| 799X | R41 | Other symptoms and signs involving cognitive functions and awareness | 2.7 |
| 781C | R26 | Abnormalities of gait and mobility | 2.6 |
| 437X | I67 | Other cerebrovascular diseases | 2.6 |
| 780D | R56 | Convulsions, not elsewhere classified | 2.6 |
| 780A | R40 | Somnolence, stupor and coma | 2.5 |
| 996H | T83 | Complications of genitourinary prosthetic devices, implants and grafts | 2.4 |
| 854B | S06 | Intracranial injury | 2.4 |
| 812A | S42 | Fracture of shoulder and upper arm | 2.3 |
| 276 | E87 | Other disorders of fluid, electrolyte and acid-base balance | 2.3 |
| 719 | M25 | Other joint disorders, not elsewhere classified | 2.3 |
| 276 | E86 | Volume depletion | 2.3 |
| 797 | R54 | Senility | 2.2 |
| V57W | Z51 | Care involving use of rehabilitation procedures | 2.1 |
| 294B | F03 | Unspecified dementia | 2.1 |
| V63X | Z75 | Problems related to medical facilities and other health care | 2.0 |
| 290E | F01 | Vascular dementia | 2.0 |
| 916 | S80 | Superficial injury of lower leg | 2.0 |
| 681 | L03 | Cellulitis | 2.0 |
| 369 | H54 | Blindness and low vision | 1.9 |
| 266C | E53 | Deficiency of other B group vitamins | 1.9 |
| V62W | Z60 | Problems related to social environment | 1.8 |
| 332A | G20 | Parkinson's disease | 1.8 |
| 780C | R55 | Syncope and collapse | 1.8 |
| 807 | S22 | Fracture of rib(s), sternum and thoracic spine | 1.8 |
| 564X | K59 | Other functional intestinal disorders | 1.8 |
| 584X | N17 | Acute renal failure | 1.8 |
| 707A | L89 | Decubitus ulcer | 1.7 |
| V02 | Z22 | Carrier of infectious disease | 1.7 |
| 041A | B95 | Streptococcus and staphylococcus as the cause of diseases classified to other chapters | 1.7 |
| 707B | L97 | Ulcer of lower limb, not elsewhere classified | 1.6 |
| 799W | R44 | Other symptoms and signs involving general sensations and perceptions | 1.6 |
| 532 | K26 | Duodenal ulcer | 1.6 |
| 458X | I95 | Hypotension | 1.6 |
| 586 | N19 | Unspecified renal failure | 1.6 |
| 038X | A41.9 | Other septicaemia | 1.6 |
| V13 | Z87 | Personal history of other diseases and conditions | 1.5 |
| 518W | J96 | Respiratory failure, not elsewhere classified | 1.5 |
| V01X | X59 | Exposure to unspecified factor | 1.5 |
| 715 | M19 | Other arthrosis | 1.5 |
| 345 | G40 | Epilepsy | 1.5 |
| 733A | M81 | Osteoporosis without pathological fracture | 1.4 |
| 821A | S72 | Fracture of femur | 1.4 |
| 808W | S32 | Fracture of lumbar spine and pelvis | 1.4 |
| 251 | E16 | Other disorders of pancreatic internal secretion | 1.4 |
| 794 | R94 | Abnormal results of function studies | 1.4 |
| 585 | N18 | Chronic renal failure | 1.4 |
| 788C | R33 | Retention of urine | 1.3 |
| 799X | R69 | Unknown and unspecified causes of morbidity | 1.3 |
| 593X | N28 | Other disorders of kidney and ureter, not elsewhere classified | 1.3 |
| 788D | R32 | Unspecified urinary incontinence | 1.2 |
| 331X | G31 | Other degenerative diseases of nervous system, not elsewhere classified | 1.2 |
| 136X | Y95 | Nosocomial condition | 1.2 |
| 959A | S09 | Other and unspecified injuries of head | 1.2 |
| 799C | R45 | Symptoms and signs involving emotional state | 1.2 |
| 435 | G45 | Transient cerebral ischaemic attacks and related syndromes | 1.2 |
| V60X | Z74 | Problems related to care-provider dependency | 1.1 |
| 729X | M79 | Other soft tissue disorders, not elsewhere classified | 1.1 |
| 873A | S01 | Open wound of head | 1.1 |
| 008W | A04 | Other bacterial intestinal infections | 1.1 |
| 009D | A09 | Diarrhoea and gastroenteritis of presumed infectious origin | 1.1 |
| 486 | J18 | Pneumonia, organism unspecified | 1.1 |
| 507 | J69 | Pneumonitis due to solids and liquids | 1.0 |
| 784F | R47 | Speech disturbances, not elsewhere classified | 1.0 |
| 268X | E55 | Vitamin D deficiency | 1.0 |
| V44 | Z93 | Artificial opening status | 1.0 |
| 785E | R02 | Gangrene, not elsewhere classified | 1.0 |
| 783X | R63 | Symptoms and signs concerning food and fluid intake | 0.9 |
| 389X | H91 | Other hearing loss | 0.9 |
| 242X | E05 | Thyrotoxicosis [hyperthyroidism] | 0.9 |
| 737D | M41 | Scoliosis | 0.9 |
| 787C | R13 | Dysphagia | 0.8 |
| V46W | Z99 | Dependence on enabling machines and devices | 0.8 |
| V09 | U82.0 | Agent resistant to penicillin and related antibiotics | 0.8 |
| 733A | M80 | Osteoporosis with pathological fracture | 0.8 |
| 536X | K92 | Other diseases of digestive system | 0.8 |
| 434X | I63 | Cerebral Infarction | 0.8 |
| 592B | N20 | Calculus of kidney and ureter | 0.7 |
| 291 | F10 | Mental and behavioural disorders due to use of alcohol | 0.7 |
| 785B | R00 | Abnormalities of heart beat | 0.7 |
| 519W | J22 | Unspecified acute lower respiratory infection | 0.7 |
| V62 | Z73 | Problems related to life-management difficulty | 0.6 |
| 790G | R79 | Other abnormal findings of blood chemistry | 0.6 |
| V15X | Z91 | Personal history of risk-factors, not elsewhere classified | 0.5 |
| 881 | S51 | Open wound of forearm | 0.5 |
| 296 | F32 | Depressive episode | 0.5 |
| 724 | M48.0 | Spinal stenosis (secondary code only) | 0.5 |
| 275 | E83 | Disorders of mineral metabolism | 0.4 |
| 716F | M15 | Polyarthrosis | 0.4 |
| 285X | D64 | Other anaemias | 0.4 |
| 686X | L08 | Other local infections of skin and subcutaneous tissue | 0.4 |
| 787A | R11 | Nausea and vomiting | 0.3 |
| 558 | K52 | Other noninfective gastroenteritis and colitis | 0.3 |
| 780G | R50 | Fever of unknown origin | 0.1 |
