# Supplementary figures and images for: Comparison of two different frailty scales in the longitudinal Swedish Adoption/Twin Study of Aging (SATSA)
Source: Scand J Public Health. 2021 Dec 14;51(4):587–94. doi: 10.1177/14034948211059958 (PMC10265292; doi:10.1177/14034948211059958)

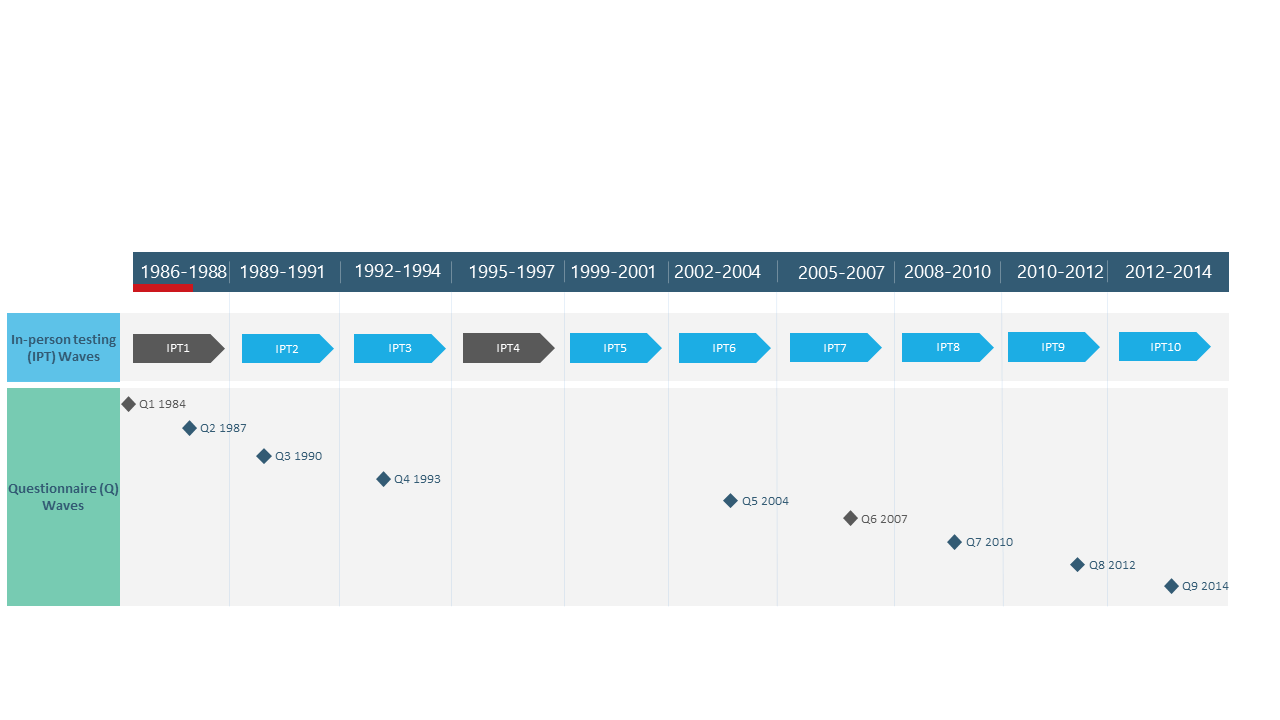

Supplement: sj-png-2-sjp-10.1177_14034948211059958 – Supplemental material for Comparison of two different frailty scales in the longitudinal Swedish Adoption/Twin Study of Aging (SATSA) [file sj-png-2-sjp-10.1177_14034948211059958.png]
